# Prevalence, distribution, enterotoxin profiles, antimicrobial resistance, and genetic diversity of *Bacillus cereus* group isolates from lettuce farms in Korea

**DOI:** 10.3389/fmicb.2022.906040

**Published:** 2022-08-23

**Authors:** Nagendran Rajalingam, Jieun Jung, Seung-Mi Seo, Hyun-Sook Jin, Bo-Eun Kim, Myeong-In Jeong, Dawoon Kim, Jae-Gee Ryu, Kyoung-Yul Ryu, Kwang Kyo Oh

**Affiliations:** ^1^Microbial Safety Division, National Institute of Agricultural Sciences, Rural Development Administration, Wanju, South Korea; ^2^Functional Food and Nutrition Division, National Institute of Agricultural Sciences, Rural Development Administration, Wanju, South Korea; ^3^Planning and Coordination Division, National Institute of Agricultural Sciences, Rural Development Administration, Wanju, South Korea

**Keywords:** *Bacillus cereus*, lettuce, toxin profiling, antimicrobial resistance, ERIC-PCR, MLST

## Abstract

Lettuce wraps are popular in Korean cuisine for their high nutritional value and versatility as healthy additions to multiple dishes. Microbial contamination of lettuce is a major concern, as lettuce is consumed fresh without cooking. Among foodborne pathogens, the spore-forming, facultative anaerobic bacterium, *Bacillus cereus* is one of the frequently detected pathogen in lettuce in Korea. In this study, we investigated the prevalence and distribution of *Bacillus cereus* strains in lettuce production farms and further evaluated the enterotoxin gene profiles, antibiotic susceptibility, multidrug resistance pattern, and genetic differences among the *B. cereus* group isolates. Of the 140 samples isolated from 10 lettuce production farms, 30 samples (21.42%) were positive for *B. cereus* in which 19 (31.6%) and 10 (23.25%) were from soil and lettuce, respectively. The enterotoxin patterns A (*hblCDA*, *nheABC*, *entFM*, and *cytK* genes) and B (*hblCDA*, *nheABC*, and *entFM* genes) accounted for 50% and 20% of all the isolates, whereas the emetic gene *cesB* was not detected in any of the *B. cereus* group isolates. Antibiotic susceptibility testing of the *B. cereus* group isolates revealed that all the strains were predominantly resistant to β-lactam antibiotics except imipenem and generally susceptible to most of the non β-lactam antibiotics, including gentamycin, streptomycin, chloramphenicol, and tetracycline. ERIC-PCR and MLST analysis revealed high genetic diversity among the 30 *B. cereus* group isolates, which belonged to 26 different sequence types (STs) and seven new STs. Moreover, isolates with identical STs exhibited similar patterns of antibiotic resistance and enterotoxin profiles. Results of this study indicate a high prevalence of *B. cereus* group isolates in lettuce production farms in the Republic of Korea.

## Introduction

Lettuce is one of the major commercial crops in Korea with a total domestic production of 86,128 tons from 3,387 ha cultivation area (Chang et al., 2020), recorded as the most cultivated leafy vegetable in Korea (Kim et al., 2018). Since lettuce is primarily consumed fresh without cooking as a wrap material or in salads, it could potentially be contaminated with pathogens across the food supply chain and may cause illness upon consumption (Bozkurt et al., 2021). The foodborne pathogens *Escherichia coli* O157:H7, *Listeria monocytogenes*, *Salmonella,* and *Bacillus cereus* are reported to be the major sources of lettuce-related outbreaks (Kim et al., 2012; Irvin et al., 2021; Qu et al., 2021). A total of 60 outbreaks related to consumption of leafy vegetables, mainly lettuce was reported to the Centers for Disease Control and Prevention (CDC) between 2014 and 2021 [Centers for Disease Control and Prevention (CDC), 2022]. Moreover, *B. cereus* was found to be the most frequently detected pathogen in fresh vegetables in Korea with a 37.5% contamination rate (Jo et al., 2011; Jung et al., 2016; Park et al., 2018). Hence, it is necessary to conduct microbial risk assessments in lettuce farms in Korea periodically and monitor the prevalence and characteristics of *B. cereus* group isolates.

The *B. cereus* group, also known as *B. cereus sensu lato,* constitutes numerous phylogenetically related species which may include *B. cereus*, *B. anthracis*, *B. thuringiensis*, *B. cytotoxicus*, *B. toyonensis, B. mycoides,* and *B. pseudomycoides* (Guinebretière et al., 2008; Liu et al., 2018; Carroll et al., 2021). These *B. cereus* group strains differ remarkably in their importance to medicine, public health and food safety (Mandic-Mulec et al., 2015). For example, some isolates of *B. cereus* and *B. cytotoxicus* strains cause foodborne illness (Naranjo et al., 2011; Guinebretière et al., 2013; Colaco et al., 2021). *B. anthracis* may cause different forms of anthrax diseases (cutaneous, gastrointestinal, and inhalational anthrax) to both animals and humans (Okinaka et al., 1999; Marston et al., 2005; Cote et al., 2015). (21)The *B. thuringiensis* strain which produces crystalline toxins is widely used in agriculture as pesticides (Bernhard et al., 1997). The psychrotolerant *B. wiedmannii* strain was reported to exhibit cytotoxic effects on vertebrate animals (Zhao et al., 2019). The probiotic strain *B. toyonensis* harbors chloramphenicol and tetracycline resistance genes *catQ* and *tet(M)* in their genome and may pose a risk for transmitting these antibiotic resistance genes to other species [EFSA Panel on Additives and Products or Substances used in Animal Feed (FEEDAP), 2012]. In addition, the *B. toyonensis* strain NCIMB 14858^T^ may cause potential risks to humans when exposed, as this strain has the capacity to produce and release functional toxins [EFSA Panel on Additives and Products or Substances used in Animal Feed (FEEDAP), 2014]. *B. pseudomycoides,* which has been recognized as a non-pathogenic environmental strain, was shown to produce pore-forming toxins and exhibit cytotoxic effects to human cells (Miller et al., 2018). The *B. weihenstephanensis* and *B. mycoides* strains, which belong to group VI phylogeny based on phylogenetic classification by Guinebretière et al. (2010) and cytotoxicity studies by Miller et al. (2018) are generally considered as low risk pathogens. The 16S rRNA gene sequences of most of the *B. cereus* group strains share 99% sequence similarity (Sacchi et al., 2002) and seem to be members of a single species (Ceuppens et al., 2013). However, they are ecologically diverse from soil to gut (Guinebretière et al., 2008; Stenfors Arnesen et al., 2008), and the pathogenic potential of these *B. cereus* group strains varies between species (Guinebretière et al., 2010). Hence, it is necessary to discriminate the *B. cereus* isolates. Combining traditional methods with molecular typing methods. Such as enterobacterial repetitive intergenic consensus–polymerase chain reaction (ERIC-PCR) and multilocus sequence typing (MLST) is recommended to discriminate diverse *B. cereus* species (Maiden et al., 2013; Zhuang et al., 2019; Qu et al., 2021).

*Bacillus cereus* is a catalase-positive, toxin-producing, and endospore-forming facultative anaerobic gram-positive bacilli present ubiquitously in soil, water, vegetables, decaying matter, and food (Osimani et al., 2018). This mesophilic pathogen can quickly proliferate at room temperature with an ample amount of preformed toxins (McDowell et al., 2021), which upon ingestion may cause gastrointestinal illness. Clinical manifestations of *B. cereus* disease include two types of gastrointestinal syndromes, i.e., diarrheal syndrome and emetic syndrome (Stenfors Arnesen et al., 2008). The diarrheal syndrome is caused by *B. cereus* strains that produce one or several enterotoxins including two tripartite complexes (Fagerlund et al., 2010), namely non-hemolytic enterotoxin (NHE, encoded by *nheA, nheB,* and *nheC*), and hemolysin BL (HBL, encoded by *hblA, hblC*, and *hblD*; Sastalla et al., 2013) and single proteins cytotoxin K (CytK; Lund et al., 2000) and enterotoxin FM (EntFM; Ngamwongsatit et al., 2008). The emetic syndrome is caused by a heat-stable toxin cerulide (Ces) preformed in the food (Carroll et al., 2017).

Contamination of lettuce can occur at any stage from farming, harvesting, processing, packing, transporting, and storing the remains by consumers (Pang et al., 2017). Several factors including the spread of feces to soil *via* farm animals, contaminated irrigation water, cross-contamination by workers from one farm to another farm may lead to disease outbreaks. Information on the prevalence and characteristics of *B. cereus* in lettuce farms will help to understand transmission routes and develop intervention technologies to ensure food safety and public health.

This study provides information about the prevalence, distribution, enterotoxin profiles and antibiotic resistance patterns of *B. cereus* group isolates in lettuce production farms. In addition, genetic diversity of the *B. cereus* group isolates was also analyzed by ERIC-PCR and MLST. The results of this study may help to develop food safety measures to reduce the contamination and transmission of *B. cereus* in lettuce farms.

## Materials and methods

### Sampling

One hundred and forty samples including lettuce (*n* = 43), soil (*n* = 60), compost (*n* = 8), and irrigation water (*n* = 29) were collected from 10 different lettuce production farms located across Jeollabuk-do province in the Republic of Korea. All the samples were aseptically placed in separate sterile bags in a cool container and transported to the laboratory and analyzed within 24 h.

### Analysis of coliforms, *Escherichia coli*, and *Bacillus cereus*

To analyze the prevalence of indicator bacteria such as aerobic bacteria, coliforms, and *E. coli*, 25 g of each sample (lettuce, soil, and compost) were homogenized in a sample bag containing 225 ml of 0.1% peptone water (PW; Oxoid, Basingstoke, United Kingdom) and homogenized in a BagMixer® (Interscience, Saint-Nom-la-Bretéche, France). The homogenized samples were tenfold serial diluted with 0.1% PW and aliquots of 1 ml were inoculated on 3 M™ Petrifilm™ Aerobic Count (AC)/Coliform Count (CC)/*E. coli* Count (EC) plates (3 M Microbiology, Minnesota, United States) and incubated at 37°C for 24 h. The irrigation water samples were evaluated for aerobic bacteria, coliforms, and *E. coli* using the Colilert-18 test kit (IDEXX laboratories, Westbrook, Maine, United States).

The prevalence of *B. cereus* was evaluated by mixing 25 g of lettuce, soil, or compost samples with 225 ml of buffered peptone water (BPW, Oxoid, Basingstoke, United Kingdom) in a stomacher bag using BagMixer® for 1 min. Tenfold serial dilutions of the homogenized samples were prepared and 0.1 ml of diluted samples were spread on Mannitol-egg yolk-polymyxin (MYP) agar plates (Oxoid, Basingstoke, United Kingdom). After incubation for 24 h at 30°C, typical pink colonies with turbid rings that generated lecithinase around them were presumptively identified as *B. cereus* and enumerated. For further confirmation, suspected colonies were sub-cultured on Tryptic Soy Agar (TSA; Difco, Sparks, MD, United States), plates, and the positive colonies were confirmed by PCR Detection Kit (Kogene Biotech, Korea).

### Distribution of enterotoxin genes

Genomic DNAs from *B. cereus* isolates were extracted using the G-spin™ bacterial genomic DNA extraction kit (iNtRON Biotechnology, Inc., Gyeonggi do, Korea) following the manufacturer’s protocol. The presence of diarrhea-causing enterotoxin genes (*nheA, nheB, nheC, hblA*, *hblC, hblD, entFM,* and *cytK*) and the emetic toxin cereulide synthesis (*cesB*) gene was determined by PCR. For PCR amplification, DNA templates of 50 ng and 10 pmol of each primer along with required distilled water (DW) were added to a PCR reaction mixture (20 μl final volume) consisting of AccuPower PCR premix (Bioneer, Daejeon, Korea) followed by amplification using the thermal cycler (C1000TM Thermal Cycler, BIORAD, CA, United States). The primer sequences and amplification procedures are described in Table 1.

**Table 1:** Primer sequences and PCR amplification procedures used in this study.

Target gene	Primer sequence	Amp.(bp)	Amplification procedure	References
*nheA*	TAC GCT AAG GAG GGG CA	499	94°C, 7 min → (94°C, 30 s → 55°C, 30 s → 72°C, 30 s) 35 cycle → 72°C, 7 min	Park et al., 2018
	GTT TTT ATT GCT TCA TCG GCT			
*nheB*	CTA TCA GCA CTT ATG GCA G	769	94°C, 7 min → (94°C, 30 s → 55°C, 30 s → 72°C, 30 s) 35 cycle → 72°C, 7 min	Park et al., 2018
	ACT CCT AGC CGG TGT TCC			
*nheC*	CGG TAG TGA TTG CTG GG	581	94°C, 7 min → (94°C, 30 s → 55°C, 30 s → 72°C, 30 s) 35 cycle → 72°C, 7 min	Park et al., 2018
	CAG CAT TCG TAC TTG CCA A			
*hblA*	GTG CAG ATG TTG ATG CCG AT	319	94°C, 7 min → (94°C, 45 s → 55°C, 45 s → 72°C, 45 s) 35 cycle → 72°C, 7 min	Park et al., 2018
	ATG CCA CTG CGT GGA CAT AT			
*hblC*	AAT GGT CAT CGG AAC TCT AT	749	94°C, 7 min → (94°C, 30 s → 55°C, 30 s → 72°C, 30 s) 35 cycle → 72°C, 7 min	Park et al., 2018
	CTC GCT GTT CTG CTG TTA AT			
*hblD*	AAT CAA GAG CTG TCA CGA AT	429	94°C, 7 min → (94°C, 30 s → 55°C, 30 s → 72°C, 30 s) 35 cycle → 72°C, 7 min	Park et al., 2018
	CAC CAA TTG ACC ATG CTA AT			
*entFM*	AAAGAAATTAATGGACAAACTCAAACTCA	596	95°C, 3 min → (95°C, 30 s → 60°C, 30 s → 72°C, 60 s) 35 cycle → 72°C, 5 min	Park et al., 2018
	GTATGTAGCTGGGCCTGTACGT			
*cytK*	GTAACTTTCATTGATGATCC	505	95°C, 1 min → (95°C, 60 s → 48°C, 60 s → 72°C, 60 s) 35 cycle → 72°C, 7 min	Park et al., 2018
	GAATACTAAATAATTGGTTTCC			
*ces*	GGTGACACATTATCATATAAGGTG	1,271	95°C, 3 min → (95°C, 60 s → 58°C, 75 s → 72°C, 50 s) 25 cycle → 72°C, 5 min	Park et al., 2018
	GTAAGCGAACCTGTCTGTAACAACA			

### Antimicrobial susceptibility testing

The antimicrobial susceptibility of *B. cereus* isolates was determined by the minimum inhibitory concentration (MIC) assay as reported by Manzulli et al. (2019) following the guidelines of the Clinical and Laboratory Standards Institute (CLSI). The antibiotics tested and concentrations used were as follows: penicillin (10 U), oxacillin (1 μg), cefotaxime (30 μg), cefoxitin (30 μg), imipenem (10 μg), gentamicin (10 μg), streptomycin (10 μg), rifampicin (5 μg), trimethoprim sulfamethoxazole (25 μg), vancomycin (30 μg), clindamycin (2 μg), erythromycin (15 μg), linezolid (30 μg), chloramphenicol (30 μg), tetracycline (30 μg), and ciprofloxacin (5 μg; Table 2). The antibiotic susceptibility of each *B. cereus* isolate was measured after incubation at 28°C for 16–18 h, and the results were interpreted following the guidelines provided by the CLSI document M100, 30th edition (CLSI, 2020). All the *B. cereus* isolates were classified as susceptible, intermediate susceptible, and resistant based on the diameter of inhibition zone and minimum inhibitory concentration (MIC) breakpoints for *Staphylococcus* spp.

**Table 2:** Antibiotics used in this study.

	Antimicrobial class	Antimicrobial agents (abbreviation) [concentration]
β-Lactams	Penicillins	Penicillin (P) [10 U], Oxacillin (OX) [1 μg]
	Cephems	Cefotaxime (CTX) [30 μg], Cefoxitin (FOX) [30 μg]
	Penems	Imipenem (IPM) [10 μg]
Non- β-lactams	Aminoglycosides	Gentamycin (CN) [10 μg], Streptomycin (S) [10 μg]
	Ansamycins	Rifampicin (RD) [5 μg]
	Folate pathway antagonists	Trimethoprim-sulfamethoxazole (SXT) [25 μg]
	Glycopeptides	Vancomycin (VA) [30 μg]
	Lincosamides	Clindamycin (DA) [2 μg]
	Macrolides	Erythromycin (E) [15 μg]
	Oxazolidinones	Linezolid (LZD) [30 μg]
	Phenicols	Chloramphenicol (C) [30 μg]
	Tetracyclines	Tetracycline (TE) [30 μg]
	-	Ciprofloxacin (CIP) [5 μg]

### Enterobacterial repetitive intergenic consensus–polymerase chain reaction

Genetic relatedness between the *B. cereus* isolates was determined by ERIC-PCR fingerprinting using the primer 5′-ATGTAAGCTCCTGGGGATTCAC-3′ designed by Versalovic et al. (1991). Amplification of repetitive ERIC sequences within the genomes of the isolates was performed with the following reaction conditions: initial denaturation at 95°C for 10 min followed by 4 cycles of 94°C for 5 min, 40°C for 5 min, 72°C for 5 min and 40 cycles of 94°C for 1 min, 55°C for 1 min, 72°C for 2 min and a final extension for 10 min at 72°C. The PCR products were evaluated by gel electrophoresis and with the Dice coefficient, the band patterns were used to generate a dendrogram by GelJ software (Heras et al., 2015) using the UPGMA (unweighted pair group method with arithmetic mean) at 1.5% optimization and tolerance level of 1.5% (Cheng et al., 2021).

### Multilocus sequence typing

To analyze the genetic diversity among the *B. cereus* isolates, genomic DNAs of the isolates were extracted and gene fragments of seven housekeeping genes [*glpF* (glycerol uptake facilitator), *gmk* (guanylate kinase), *ilvD* (dihydroxy-acid dehydratase), *pta* (phosphate acetyltransferase), *pur* (phosphoribosylaminoimidazolecarboxamide), *pycA* (pyruvate carboxylase), and *tpi* (triosephosphate isomerase)] were amplified using the primers and conditions listed in the MLST database (Jolley et al., 2018).^1^ The resulting DNA fragments were purified and sequenced using an automated DNA analyzer (ABI 3730XL; Applied Biosystems, Foster City, CA) by SolGent Korea (SolGent, Daejeon, Korea). With the sequence information, allele numbers for each housekeeping gene were assigned based on the locus queries from the MLST database^2^ and the sequence types (STs) were determined based on the numeric allelic profile of the isolates. The genetic relationships among the *B. cereus* isolates were determined using the Global Optimal eBURST (goeBURST) algorithm (Francisco et al., 2009) implemented in the PHYLOViZ software (Ribeiro-Gonçalves et al., 2016; Liu et al., 2017).

## Results and discussion

### Prevalence of coliforms, *Escherichia coli*, and *Bacillus cereus* in lettuce production farms

The prevalence and distribution of aerobic bacteria, coliforms, *E. coli,* and *B. cereus* in the lettuce, soil, compost, and irrigation water samples collected from 10 different farms are presented in Table 3. Of the total 140 samples, aerobic bacteria and coliforms were more prevalent in soil (6.89 ± 0.19 log CFU/g; 3.07 ± 0.36 log CFU/g) and compost (5.44 ± 0.1 log CFU/g; ND) followed by lettuce (5.12 ± 0.37 log CFU/g; 2.43 ± 0.23 log CFU/g), while the irrigation water (2.18 ± 0.18 log CFU/ml; 0.32 ± 0.12 log CFU/ml) was least contaminated with indicator bacteria. *E. coli* was detected in lettuce samples from farms 2 and 3 and soil sample from farm 4, while *E. coli* was not detected in all the other samples.

**Table 3:** Population density of indicator bacteria and *Bacillus cereus* in lettuce and its production environments.

Farm	Sample	No. of samples	Aerobic bacteria (log CFU/g)^*^	Coliform (log CFU/g)^*^	*Escherichia coli* (log CFU/g)^*^	*Bacillus cereus* (log CFU/g)^*^	*Bacillus cereus group* isolates (%) (groEL + gyrB)
Farm 1	Lettuce	6	3.47 ± 0.56	ND	ND	1.48	ND
	Soil	6	5.37 ± 0.71	2.21 ± 0.32	ND	4.38 ± 0.69	3/6 (50%)
	Compost	3	4.97 ± 0.17	ND	ND	2.48 ± 0.0	1/3 (16.7%)
	Irrigation water	3	1.14 ± 0.22	ND	ND	ND	ND
Farm 2	Lettuce	11	4.58 ± 0.41	1.74 ± 0.27	1.71 ± 0.31	2.50 ± 0.99	3/11 (27.2%)
	Soil	12	6.86 ± 0.23	2.65 ± 0.42	ND	3.79 ± 0.60	6/12 (50%)
	Compost	1	6.06 ± 0	ND	ND	ND	ND
	Irrigation water	6	1.72 ± 0.10	ND	ND	ND	ND
Farm 3	Lettuce	5	5.99 ± 0.72	2.17 ± 0.27	1.80 ± 0.35	1.87 ± 0.12	ND
	Soil	15	7.34 ± 0.19	2.59 ± 0.35	ND	5.78 ± 0.28	1/15 (6.7%)
	Compost	1	4.38 ± 0	ND	ND	4.14 ± 0.23	ND
	Irrigation water	3	3.35 ± 0.16	0.05 ± 0.08	ND	ND	ND
Farm 4	Lettuce	3	7.71 ± 0.25	2.63 ± 0.57	ND	2.39 ± 0.74	1/3 (16.7%)
	Soil	9	7.21 ± 0.11	2.90 ± 0.37	2.28 ± 0.62	ND	3/9 (33.3%)
	Compost	3	6.37 ± 0.21	ND	ND	ND	ND
	Irrigation water	3	1.62 ± 0.09	ND	ND	ND	ND
Farm 5	Lettuce	3	5.02 ± 0.25	2.24 ± 0	ND	1.4 ± 0.2	1/3 (16.7%)
	Soil	3	7.23 ± 0.11	3.74 ± 0.43	ND	4.31 ± 0.08	ND
	Irrigation water	2	3.22 ± 0.46	0.33 ± 0.21	ND	1.21 ± 0.07	ND
Farm 6	Lettuce	3	5.18 ± 0.15	ND	ND	2.21 ± 0.7	1/3 (16.7%)
	Soil	3	6.78 ± 0.11	ND	ND	4.6 ± 0.21	2/3 (66.7%)
	Irrigation water	2	2.62 ± 0.22	0.58 ± 0.08	ND	1.05 ± 0.07	ND
Farm 7	Lettuce	3	4.35 ± 0.5	ND	ND	1.34 ± 0.73	ND
	Soil	3	7.16 ± 0.14	ND	ND	4.18 ± 0.14	ND
	Irrigation water	2	1.75 ± 0	ND	ND	0	ND
Farm 8	Lettuce	3	6.04 ± 0.22	ND	ND	1.33 ± 0.52	1/3 (16.7%)
	Soil	3	7.21 ± 0.03	ND	ND	4.92 ± 0.06	ND
	Irrigation water	2	3.38 ± 0.06	ND	ND	1.85 ± 0.1	ND
Farm 9	Lettuce	3	4.72 ± 0.46	2.77 ± 0.02	ND	1.64 ± 0.28	1/3 (16.7%)
	Soil	3	6.86 ± 0.19	4.37 ± 0.28	ND	5.2 ± 0.38	2/3 (66.7%)
	Irrigation water	3	0.83 ± 0.29	ND	ND	ND	ND
Farm 10	Lettuce	3	4.17 ± 0.17	3.08 ± 0.29	ND	ND	ND
	Soil	3	6.87 ± 0.09	3.06 ± 0.35	ND	4.58 ± 0.07	2/3 (66.7%)
	Irrigation water	3	ND	ND	ND	ND	ND

* The data represented here are the mean values of the samples.

Preliminary evaluation of *B. cereus* contamination analysis revealed that soil (4.63 ± 0.28 log CFU/g) and compost (3.31 ± 0.12 log CFU/g) was more prone to *B. cereus* contamination than lettuce (1.79 ± 0.64 log CFU/g) and irrigation water (1.03 ± 0.06 log CFU/ml). However, qualitative assessment results revealed that 28 out of 140 samples (20%) were contaminated with *B. cereus* group isolates, in which 19 isolates were from soil (31.6%), 8 from lettuce (18.60%), and 1 isolate from compost (12.50%). No *B. cereus* group strains were detected in irrigation water (Table 3).

### Profiling of enterotoxin genes among *Bacillus cereus* isolates

Identifying and profiling toxin genes of *B. cereus* group strains is an important step in characterizing the virulence potential of the *B. cereus* isolates mainly distinguishing the pathogenic potential of the strains. In this study, genomic DNA of 30 *B. cereus* isolates from lettuce, soil and compost samples from various farms were extracted, and the prevalence of enterotoxin genes *nheA, nheB, nheC, hblA, hblC, hblD, entFM,* and *cytK* and the emetic gene *ces* were determined by PCR. The distribution of enterotoxin genes among *B. cereus* isolates is summarized in Table 4. All the *B. cereus* isolates carried at least one gene encoding NHE toxin including *nheA* (29 out of 30 isolates, 96.6%), *nheB* (24 out of 30, 80%), *nheC* (29 out of 30, 97%), and at least one HBL toxin encoding gene including *hblA* (23 out of 30, 76.6%), *hblC* (28 out of 30, 93.3%), and *hblD* (29 out of 30, 97%). All the isolates except B18 possessed *entFM* (29 out of 30, 97%). The dermonecrotic *cytK* gene was detected in 19 out of 30 (63.3%) *B. cereus* group isolates (Table 4). However, the emetic gene *ces* was not detected in any of the *B. cereus* group isolates.

**Table 4:** Distribution of toxin genes among *Bacillus cereus* strains isolated from lettuce farms.

No.	Strain	Farms	Source	*hbl* complex			*nhe* complex			*entFM*	*cytK*	*ces*	Enterotoxin profile
				*hblA*	*hblC*	*hblD*	*nheA*	*nheB*	*nheC*				
1	B11	Farm 1	Soil	+	+	+	+	+	+	+	+	−	A
2	B12-1	Farm 1	Soil	−	+	+	+	+	+	+	+	−	C
3	B18	Farm 1	Compost	+	+	+	+	+	+	+	+	−	A
4	B20-5	Farm 3	lettuce	+	+	+	−	−	+	+	+	−	D
5	B24-5	Farm 3	lettuce	+	+	+	+	+	+	+	−	−	B
6	B28-2	Farm 3	Soil	+	+	+	+	+	+	+	+	−	A
7	B29-2	Farm 3	Soil	+	+	+	+	+	+	+	−	−	B
8	B52	Farm 2	Lettuce	+	+	+	+	+	+	+	+	−	A
9	B56-1	Farm 2	Soil	−	−	+	+	+	+	+	+	−	E
10	B60-1	Farm 2	Soil	+	+	+	+	+	+	+	−	−	B
11	B61-1	Farm 2	Soil	−	+	−	+	−	+	+	+	−	F
12	B79-1	Farm 4	Soil	+	+	+	+	+	+	+	+	−	A
13	B8-1	Farm 1	Soil	+	+	+	+	+	+	+	+	−	A
14	B89	Farm 3	Lettuce	+	+	+	+	+	+	+	+	−	A
15	B90-2	Farm 3	Soil	+	+	+	+	+	+	+	+	−	A
16	B93-1	Farm 3	Soil	+	+	+	+	+	+	+	−	−	B
17	B94-1	Farm 3	Soil	+	+	+	+	+	+	+	+	−	A
18	B95-1	Farm 3	Soil	+	+	+	+	+	+	+	−	−	B
19	102–2	Farm 5	Lettuce	+	+	+	+	+	+	+	+	−	A
20	110–1	Farm 6	Lettuce	+	+	+	+	−	+	+	−	−	G
21	112–1	Farm 6	Soil	+	+	+	+	+	+	+	+	−	A
22	113–1	Farm 6	Soil	+	+	+	+	+	+	+	+	−	A
23	126–1	Farm 7	Lettuce	+	+	+	+	+	+	+	+	−	A
24	B8	Farm 8	Lettuce	+	+	+	+	+	+	+	+	−	A
25	B11-1	Farm 8	Soil	+	+	+	+	+	+	+	+	−	A
26	B12-1	Farm 8	Soil	+	+	+	+	+	+	+	−	−	B
27	B17	Farm 8	Lettuce	−	+	+	+	−	−	+	−	−	H
28	B18	Farm 8	Lettuce	−	−	+	+	−	+	−	−	−	I
29	B20-2	Farm 8	Soil	−	+	+	+	+	+	+	−	−	J
30	B21-1	Farm 8	Soil	−	+	+	+	−	−	+	−	−	H
Rate (%)				77%	93%	97%	97%	80%	93%	97%	63%	0%	

+, Positive; −, Negative.

The *B. cereus* isolates were further classified into 11 groups (enterotoxin profile patterns A to K) based on the presence or absence of 8 enterotoxin genes and 1 emetic gene (Table 5). The enterotoxin patterns A (*hblCDA, nheABC, entFM*, and *cytK* gene) and B (*hblCDA, nheABC,* and *entFM* gene) accounted for 50% (15/30) and 20% (6/30) of all the isolates, while the remaining patterns, C to K were distributed evenly among the remaining 9 isolates, sharing 3.33% (1/30) each, i.e., 1 pattern per isolate. Corresponding to our observations, Kim et al. (2011) has also reported that the majority of their *B. cereus* isolates (79.2%) possessed enterotoxin profile patterns I and II which are patterns A and B in our study (Kim et al., 2011). Moreover, the *B. cereus* group strains with profile A containing all the enterotoxin genes are critically important strains among the environment, food, and clinical isolates (Hsu et al., 2021).

**Table 5:** Enterotoxin gene profiles of *Bacillus cereus* group isolates.

Profile	Enterotoxin genes									No. of samples
	*hblA*	*hblC*	*hblD*	*nheA*	*nheB*	*nheC*	*entFM*	*cytK*	*ces*	
A	(+)	(+)	(+)	(+)	(+)	(+)	(+)	(+)	(−)	15
B	(+)	(+)	(+)	(+)	(+)	(+)	(+)	(−)	(−)	6
C	(−)	(+)	(+)	(+)	(+)	(+)	(+)	(+)	(−)	1
D	(+)	(+)	(+)	(−)	(−)	(+)	(+)	(+)	(−)	1
E	(−)	(−)	(+)	(+)	(+)	(+)	(+)	(+)	(−)	1
F	(−)	(+)	(−)	(+)	(−)	(+)	(+)	(+)	(−)	1
G	(+)	(+)	(+)	(+)	(−)	(+)	(+)	(+)	(−)	1
H	(−)	(+)	(+)	(+)	(−)	(−)	(+)	(−)	(−)	2
I	(−)	(−)	(+)	(+)	(−)	(+)	(−)	(−)	(−)	1
J	(−)	(+)	(+)	(+)	(+)	(+)	(+)	(−)	(−)	1
Total										n = 30

(+) represents successful amplification of target genes by multiplex PCR (hbl and nhe complex genes) and singleplex PCR (*entFM*, *cytK*, and *ces*).

Since the major virulence factors hemolytic (HBL) and non-hemolytic (NHE) enterotoxins are three-component toxin complexes (Stenfors Arnesen et al., 2008), all the three genes should be present in the *nheABC* and *hblCDA* clusters to generate active toxins in both cases (Chaves et al., 2011; Sastalla et al., 2013). Based on these criteria, only 76% and 80% of the *B. cereus* isolates produce active form of HBL and NHE enterotoxins, respectively, and 70% of isolates which possess all the genes in both the clusters are highly pathogenic and may cause diarrhea (Table 4). The enterotoxin *entFM*, a cell wall peptidase necessary for bacterial morphology, motility, cell adhesion, and biofilm formation (Darrigo et al., 2016; Tran et al., 2020) is prevalent in the genome of almost all the members of the *B. cereus* group (Kim et al., 2011; Chon et al., 2015) which is consistent with our observation. The absence of emetic toxin gene *ces* in all strains indicates that the enterotoxin gene containing *B. cereus* strains are widespread in Korea (Kim et al., 2009) than the emetic strains as reported by Kim et al. (2011).

### Antimicrobial susceptibility of *Bacillus cereus* isolates

Antibiotic resistance or susceptibility of *B. cereus* isolates to different antimicrobial agents is represented in Table 6. All the *B. cereus* isolates, regardless of the source (lettuce, soil, and compost) were resistant to penicillin (100%), oxacillin (100%), cefoxitin (100%), and cefotaxime (77% with 33% intermediate resistance). In contrast, all the *B. cereus* isolates were susceptible to imipenem (100%), gentamycin (100%), streptomycin (100%), trimethoprim-sulfamethoxazole (100%), ciprofloxacin (100%), chloramphenicol (100%), linezolid (100%), vancomycin (97%), erythromycin (93%) and tetracycline (93%). The ansamycin class antibiotic rifampicin inhibited the growth of 50% of the *B. cereus* isolates, while 33% showed intermediate resistance and the remaining 17% of isolates were resistant toward rifampicin. In the case of clindamycin, 60% of isolates were sensitive and 40% of strains showed intermediate resistance.

**Table 6:** Antibiotic susceptibility of *Bacillus cereus* group isolates.

Antimicrobial agents	Lettuce (*n* = 10)	Soil (*n* = 19)	Compost (*n* − 1)
	R, I	R, I	R, I
Penicillin (P)	10 (100%); 0 (0%)	19 (100%); 0(0)%	1 (100%); 0 (0%)
Oxacillin (OX)	10 (100%); 0 (0%)	19 (100%); 0(0)%	1 (100%); 0 (0%)
Cefotaxime (CTX)	7 (70%); 4 (40%)	15 (78.9%); 3 (15.7%)	1 (100%); 0 (0%)
Cefoxitin (FOX)	10 (100%); 0 (0%)	19 (100%); 0(0)%	1 (100%); 0 (0%)
Imipenem (IPM)	0 (0%); 0(0%)	0 (0%); 0(0%)	0 (0%); 0(0%)
Gentamycin (CN)	0 (0%); 0(0%)	0 (0%); 0(0%)	0 (0%); 0(0%)
Streptomycin (S)	0 (0%); 0(0%)	0 (0%); 0(0%)	0 (0%); 0(0%)
Rifampicin (RD)	2 (20); 3 (3%)	2 (10.5%); 6 (31.5%)	0 (0%); 1 (100%)
Vancomycin (VA)	0 (0%); 0(0%)	0 (0%); 0(0%)	0 (0%); 0(0%)
Clindamycin (DA)	0 (0%); 1 (10%)	0 (0%); 0(0%)	0 (0%); 0(0%)
Erythromycin (E)	0 (0%); 4 (40%)	0 (0%); 8 (42.1%)	0 (0%); 0(0%)
Linezolid (LZD)	0 (0%); 1 (10%)	0 (0%); 1 (5.26%)	0 (0%); 0(0%)
Chloramphenicol (C)	0 (0%); 0(0%)	0 (0%); 0(0%)	0 (0%); 0(0%)
Tetracycline (TE)	0 (0%); 0(0%)	0 (0%); 0(0%)	0 (0%); 0(0%)
Ciprofloxacin (CIP)	1 (10%); 0 (0%)	0 (0%); 1 (5.26%)	0 (0%); 0(0%)

R, resistance; I, intermediate.

Pathogens develop antibiotic resistance through several mechanisms, including the acquisition of resistance determinants *via* horizontal gene transfer from soil and environmental bacteria (Munita and Arias, 2016; Peterson and Kaur, 2018) or by a random mutation generated by a consequence of antibiotic selection pressure, allowing them to survive and proliferate in the presence of the antimicrobial agent (Skalet et al., 2010). Since *B. cereus* group strains are clinically significant, determining their resistance/susceptibility toward antimicrobial agents is essential for treatment during disease outbreaks. Even though most infections caused by *B. cereus* group strains may get better without antimicrobial treatment, it is important to be vigilant as new *B. cereus* group infection strains may emerge in the future (Mills et al., 2022). Based on the antibiotic susceptibility profiles, the *B. cereus* group isolates were further classified into four different groups (I, II, III, and IV) according to the MDR patterns (Table 7).

**Table 7:** Multidrug resistance (MDR) patterns of *Bacillus cereus* group isolates.

MDR pattern	MDR profile	No. (%) of isolates (*n* = 30)	Source and no. of isolates (*n* = 30)
I	P, OX, FOX	7 (23.3%)	L: 3, S: 4, C: 0, IW: 0
II	P, OX, CTX, FOX	19 (63.3%)	L: 5, S: 13, C: 1, IW: 0
III	P, OX, CTX, FOX, RD	3 (30%)	L: 1, S: 2, C: 0, IW: 0
IV	P, OX, CTX, FOX, RD, TE	1 (3.3%)	L: 1, S: 0, C: 0, IW: 0

P, Penicillin; OX, Oxacillin; FOX, Cefoxitin; CTX, Cefotaxime; RD, Rifampicin; TE, Tetracycline.

Most of the *B. cereus* isolates (63.3%) were resistant to all the penicillins (P, OX) and cephems (CTX, FOX) class β-lactam antibiotics falling under pattern II while 23.3% of isolates were resistant to P, OX, and FOX (pattern I) but not to CTX. Generally, all the *Bacillus cereus* group strains are predominantly resistant to β-lactam antibiotics due to the abundant production of β-lactamase enzymes (Majiduddin et al., 2002; Owusu-Kwarteng et al., 2017). Correspondingly, all the *B. cereus* isolates in our study were resistant to β-lactam antibiotics except imipenem which showed 100% susceptibility toward *B. cereus* isolates (Table 7). This was due to the strong inhibition efficiency of the carbapenem antibiotic imipenem against β-lactamases (Vardakas et al., 2012). According to the results of our study, the *B. cereus* isolates were generally susceptible to most of the non-β-lactam antibiotics including gentamycin, streptomycin, trimethoprim-sulfamethoxazole, ciprofloxacin, chloramphenicol, linezolid, vancomycin, erythromycin, and tetracycline, which is consistent to the reports of other studies (Luna et al., 2007; Fiedler et al., 2019). However, the *B. cereus* isolates in our study showed an intermediate level of resistance toward rifampicin and clindamycin which has also been reported earlier in other studies (Park et al., 2009, 2018, 2020).

### Genetic diversity analysis of *Bacillus cereus* group isolates by ERIC-PCR and MLST

**Figure 1:**
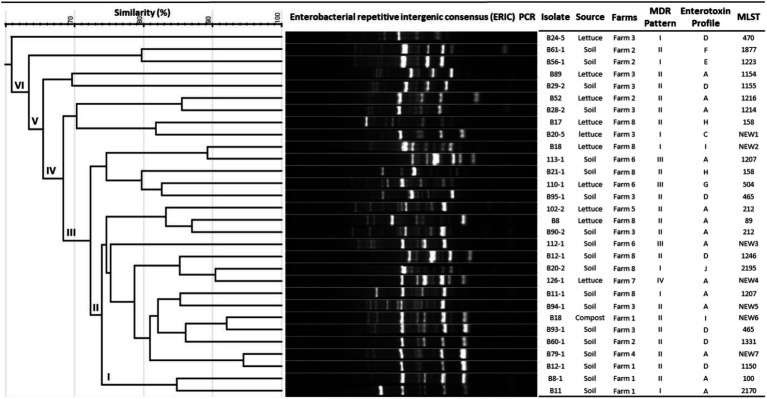
Genetic diversity analysis based on ERIC-PCR and MLST and their correlation with MDR patterns and enterotoxin profiles of *Bacillus cereus* isolates.

Genomic fingerprinting by ERIC-PCR has been successfully used to analyze the genetic diversity of several bacteria and discriminate between closely related strains. The combined results of ERIC-PCR fingerprinting, MLST, enterotoxin profiles, and multidrug resistance patterns of *B. cereus* group isolates are summarized in Figure 1. ERIC-PCR fingerprinting of 30 *B. cereus* isolates from lettuce farms yielded 6 to 12 polymorphic bands ranging in size between 100 to over 3,000 base pairs (bp) with different band intensities. The most common molecular sizes of the bands in the ERIC-PCR gel image were around 300, 450, 600, and 900 bps. Based on the migration patterns of the PCR products, the *B. cereus* isolates were classified into 6 different groups (designated as I, II, III, IV, V, and VI) by UPGMA clustering. All the isolates from farms 1, 4, and 7 were within cluster I, while isolates from farms 6 and 8 were distributed within clusters I, II, and III, indicating that all these isolates are genetically related. The presence of *B. cereus* isolates from farms 2 and 3 in all the clusters shows their genetic diversity. However, the ERIC-PCR-based clustering did not correlate with the sources of the isolates or MDR patterns or enterotoxin profiles of the isolates (Figure 1).

MLST-based genetic subtyping works by indexing the variation of nucleotide sequences in multiple housekeeping gene (loci) fragments distributed around the bacterial genome. Based on the nucleotide sequence variations, a numeric integer is designated to each locus and an allelic profile is created and with a unique combination of alleles, a sequence type (ST) number is assigned (Jolley and Maiden, 2014) with which the genetic diversity of the bacterial isolates can be characterized. In our study, MLST analysis of 30 *B. cereus* isolates resulted in identifying 26 STs from PubMLST database and 7 new STs.

**Figure 2:**
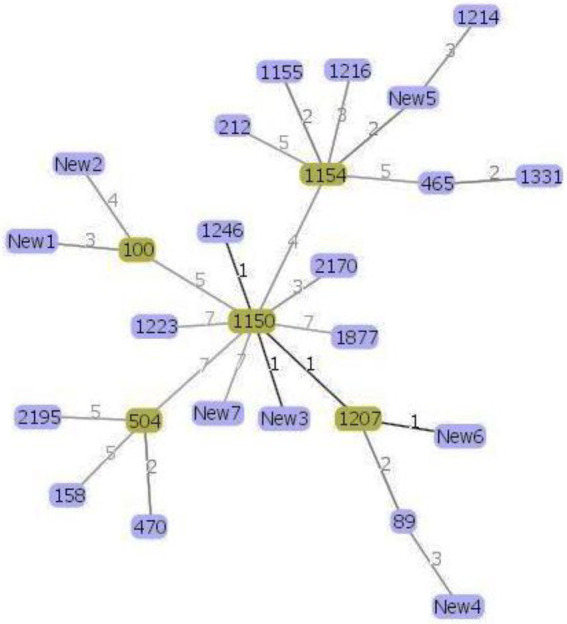
Genomic relatedness between the 30 *Bacillus cereus* isolates based on MLST data. The minimum spanning tree was constructed using the goeBURST algorithm implemented in the PHYLOViZ online platform. Green color represents the subgroup founders while single-locus variants are highlighted with darker links.

Phylogenetic analysis helps us to better understand the evolutionary relationship between the organisms and thus helps us to characterize newly evolved isolates (Soltis and Soltis, 2003). MLST data were used to infer possible phylogenetic relationship between the isolates using the goeBURST algorithm (Francisco et al., 2009; Pérez-Losada et al., 2013). The goeBURST analysis revealed a close relationship between the soil isolate ST1150 and three other STs ST1246, ST1207, and a new ST New3 isolated from soil and between ST1207 and the novel ST New6 (Figure 2) isolated from lettuce. These STs pairs are single locus variants (SLVs) that differed from one another by one housekeeping gene allele.

Moreover, according to the PubMLST database, the soil and lettuce isolates ST1214, ST1216, and ST1223 from our study were previously isolated from food packing materials in China; ST1150, ST1155, and ST1154 were previously isolated from soil samples from Japan; ST1207 and ST1246 were separated from feces samples from Korea and ST465 as previously isolated from Taiwan. The ST158 strain isolated from soil in this study was previously reported in isolates from Korea, Japan, and United States. ST100 was reported as blood isolate and milk product isolate from the USA and China, respectively. Moreover, other isolates from the USA included ST2195, ST89, ST504, and ST470. The soil isolates ST1223 and ST212 were identified in PubMLST database as isolates from unknown sources. Out of 7 novel STs, 1 isolate was from compost and 3 isolates each were from soil and lettuce samples. These results suggest that *B. cereus* isolates found in lettuce farms may be acquired through several sources. Furthermore, the results of MLST coincided with the patterns of antibiotic resistance and enterotoxin profiles of the isolates (Figure 1) and thus confirmed the genetic diversity within the *B. cereus* isolates.

## Conclusion

In this study, we investigated the prevalence and distribution of *B. cereus* group isolates in 10 lettuce farms located in Korea and analyzed their toxin profiles, antibiotic resistance patterns, and genetic diversity among the isolates by ERIC-PCR and MLST. Thirty *B. cereus* group isolates were identified in 140 samples from lettuce farms. The enterotoxin patterns A (*hblCDA, nheABC, entFM*, and *cytK* gene) and B (*hblCDA, nheABC,* and *entFM* gene) accounted for 50% and 20% of all the isolates whereas the emetic gene *ces* was not detected in any of the *B. cereus* group isolates. The presence of enterotoxin genes in all the isolates indicates that the enterotoxin gene containing *B. cereus* strains are widespread in Korea than the emetic strains. Antibiotic susceptibility testing revealed that the *B. cereus* isolates in our study were predominantly resistant to β-lactam antibiotics except imipenem and generally susceptible to most of the non β-lactam antibiotics including gentamycin, streptomycin, trimethoprim-sulfamethoxazole, ciprofloxacin, chloramphenicol, linezolid, vancomycin, erythromycin, and tetracycline. Genetic fingerprinting by ERIC-PCR and MLST analysis revealed that high genetic diversity is prevalent among the *B. cereus* strains isolated from the lettuce farms in Korea. The results of this study provide information about the contamination levels and molecular characteristics of *B. cereus* group strains isolated from different lettuce farms which may help to improve the microbial safety standards in lettuce farms.

## Data availability statement

The raw data supporting the conclusions of this article will be made available by the authors, without undue reservation.

## Author contributions

NR performed some experiments, analyzed majority of the data, and wrote the whole manuscript. JJ, S-MS, and H-SJ prepared all the necessary materials and performed most of the experiments. B-EK and M-IJ contributed to the discussion of experimental results. J-GR, K-YR, and KO designed and supervised the experiments at different stages. All authors contributed to the article and approved the submitted version.

## Funding

This research was funded by “Research Program for Agricultural Science and Technology Development (project no. PJ015168022022),” National Institute of Agricultural Sciences, Rural Development Administration, South Korea.

## Conflict of interest

The authors declare that the research was conducted in the absence of any commercial or financial relationships that could be construed as a potential conflict of interest.

## Publisher’s note

All claims expressed in this article are solely those of the authors and do not necessarily represent those of their affiliated organizations, or those of the publisher, the editors and the reviewers. Any product that may be evaluated in this article, or claim that may be made by its manufacturer, is not guaranteed or endorsed by the publisher.

## Footnotes

^1^ https://pubmlst.org/organisms/bacillus-cereus/primers

^2^ https://pubmlst.org/bcereus/
